# Fiber-Coupled Multipass NIR Sensor for In Situ, Real-Time Water Vapor Outgassing Monitoring

**DOI:** 10.3390/s25123824

**Published:** 2025-06-19

**Authors:** Logan Echeveria, Yue Hao, Michael C. Rushford, Gerardo Chavez, Sean Tardif, Allan Chang, Sylvie Aubry, Maxwell Murialdo, J. Chance Carter, Brandon Foley, Pratanu Roy, S. Roger Qiu, Tiziana Bond

**Affiliations:** Lawrence Livermore National Laboratory, Livermore, CA 94550, USA; echeveria1@llnl.gov (L.E.); sylvie.aubry@llnl.gov (S.A.);

**Keywords:** optical multipass sensor, gas sensor, material outgassing, water vapor monitoring

## Abstract

This work presents the recent development of a fiber-coupled multipass near-infrared (NIR) gas sensor used to monitor water vapor desorption of small material coupons. The gas sensor design employs a White cell topology to maximize the optical path length over a compact, hand-size footprint. Water vapor concentrations are quantified over a large dynamic range by simultaneously applying wavelength modulation and tunable diode laser absorption spectroscopy techniques. A custom headspace optimized for material desorption experiments is assembled using commercially available vacuum chamber components. We provide in situ measurements of water vapor desorption from two geometries of the industrially important silicone elastomer Sylgard-184 as a case study for sensor viability. To corroborate the results, the gas sensor data are compared to numerical simulations based on a triple-mode diffusion–sorption model, consisting of Henry, Langmuir, and Pooling modes.

## 1. Introduction

The uptake and desorption of water vapor is a critical phenomenon to study for accurately characterizing the lifetime of materials. In closed systems such as satellites, batteries, and food packaging, water vapor outgassing from one material can create conditions that accelerate aging in other components. This can lead to issues such as metal corrosion, mold or fungal growth causing biodegradation, and the chemical breakdown of polymers [1,2,3]. Failure to properly characterize material lifetimes or to understand how materials in a closed system interact can result in significant health, safety, and economic consequences [4].

Traditionally, testing a material’s shelf life in a shared headspace involves a series of independent trials where outgassing constituents of samples are first measured by themselves (aging) followed by additional trials pairing materials in the system together (compatibility). Typically, deployed benchtop spectroscopy tools such as gas chromatography mass-spectroscopy (GC-MS) are accurate but are large-scale instruments that have slow response times (hours) making them difficult to integrate for in situ analysis [5,6]. In situ gas analysis is desirable in order to assess the transient response of a material’s gas desorption profile, giving insight into important material properties such as the diffusivity [7]. Techniques, such as gravimetric analysis, can measure vapor uptake and desorption in situ, but require expensive equipment to precisely monitor the sample’s mass (accuracy < 0.1 µg typically required) and to control the environment’s temperature and humidity [8]. Furthermore, completing an exhaustive compatibility assessment for all material pairings in a system is a lengthy process and therefore should incorporate analysis tools that are easily parallelizable. Additionally, the selection of materials for a system is often a fast-paced process mainly driven by advancements in manufacturing of next generation materials such as 3D-printed elastomers [9]. Therefore, it is critical to provide flexible systems for real-time measurements for assessing how materials age and influence other components a given system.

Gas sensors based on optical absorption are especially attractive due to their ultrafast response times, high analyte specificity and sensitivity based on the spectral absorbance band, and resistance to harsh environments [10,11]. Tunable diode laser absorption spectroscopy (TDLAS) is a widely adopted optical analysis technique that offers a straightforward, yet sensitive, method of interrogating a gas species. Based on Beer–Lambert’s law, TDLAS relates the optical path length, strength of the analyte’s absorption line, and absorbance measured by the optical sensor to the analyte’s concentration [12]. The user can select any absorption line to interrogate so long as a diode that matches the wavelength is readily available. Wavelength modulation spectroscopy (WMS) is a complementary analytical technique that greatly enhances the limit of detection of direct absorption spectroscopy [13]. This technique superimposes a high frequency sine wave onto the slower TDLAS scan across an analyte’s absorption line. Utilizing these two techniques in tandem allows for a sensitive method for measuring gas species over a large dynamic range without the need for bulky equipment that other optical analysis techniques call for such as Fourier-transform infrared spectroscopy.

In this study, we demonstrate new fabrication and experimental protocols for deployment of a custom multipass near infrared (NIR) optical sensor that uses TDLAS and WMS to monitor water vapor desorption in situ and in real-time within small volumes. We first report on the development of a custom-built, compact optical NIR spectroscopic sensor that can be used to interrogate a headspace during moisture diffusion studies. We discuss the procedure for embedding the sensor into a sealed mechanical assembly specifically designed for in situ material outgassing experiments. A key consideration for in situ sensor deployment is to ensure the sensor being introduced into the headspace does not produce unwanted constituents that could interfere with the materials being studied. Our implementation of the White cell concept uses an epoxy-free, all-mechanical design to make the sensor inert and agnostic to the headspace conditions. We then describe the experimental workflow for moisture uptake and outgassing of Sylgard-184, a commercially available silicone elastomer employed in electronic encapsulation, optical applications, microfluidic device fabrication, and protective coatings due to its excellent dielectric properties, optical clarity, and mechanical flexibility. The water vapor outgassing profile is then compared to a reaction–sorption–transport modeling tool (previously developed by members at Lawrence Livermore National Laboratory) to cross-validate the steady state and transient response of the material outgassing profile [8].

## 2. Materials and Methods

**Figure 1:**
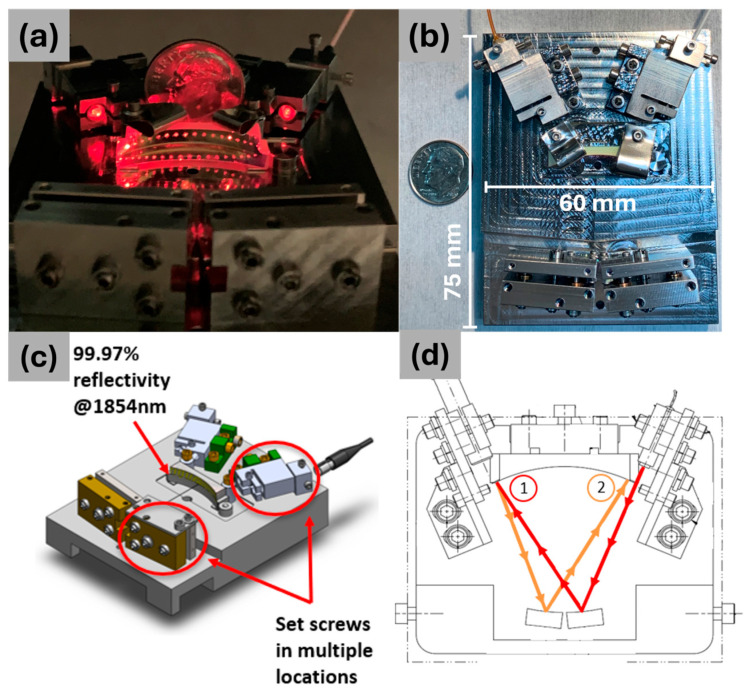
(**a**) Photo of the multipass sensor with a 633 nm laser used as the input to visualize the optical path. (**b**) Top view of the sensor layout. (**c**) CAD model of the sensor with notable set screws circled in red. (**d**) First two passes of the optical path in a White cell geometry.

Our optical sensor utilizes a White cell multipass configuration to enhance optical path length and sensitivity. The concept, first published in 1942 by White, arranges three mirrors of equivalent radii of curvature in a triangle formation at a distance equal to their radii of curvature [14]. The geometry allows for a long optical path to be folded many times (visualized in Figure 1d), thus maximizing the light–matter interaction over a constant footprint. The all-mechanical design incorporates stainless-steel components that secure the mirrors and optical fibers on a stainless-steel base plate. Two cylindrical mirrors, each with a diameter of 6 mm and radius of curvature of 25.4 mm, are positioned inside a dual-plate housing unit (Figure 1b). The design incorporates set screws located throughout the assembly, enabling minor adjustments or major realignment procedures (Figure 1c). All mechanical parts for the multipass cell were fabricated by Proto Labs, Incorporated, Maple Plain, MN, USA. The custom-made mirrors, supplied by Optimax Systems, Incorporated, Ontario, NY, USA were coated with a thin-film dielectric layer to maximize reflection at 633 nm for visual alignment and 1854 nm for water absorption (coating reflection spectra are shown in Figure S1a). Using an NIR wavelength for water vapor absorption aligns with the focus of this study and has the added benefit of enabling the use of readily available and inexpensive optical components and coatings. The water vapor absorption band at 1854 nm was selected due to its high overtone/combination absorption band and low background noise (the absorption profile for water vapor from HITRAN is shown in Figure S1b).

Each red spot on the large mirror shown in Figure 1a represents 5.08 cm of optical path length. In this image, the total optical path length is calculated to be 1.83 m. The total path length can be tuned to increase the total optical path length with the tradeoff of decreasing the transmission through the sensor. For this work, a path length of 1.83 m was sufficient to accurately assess the dynamic range of anticipated water vapor concentration from the material coupons.

**Figure 2:**
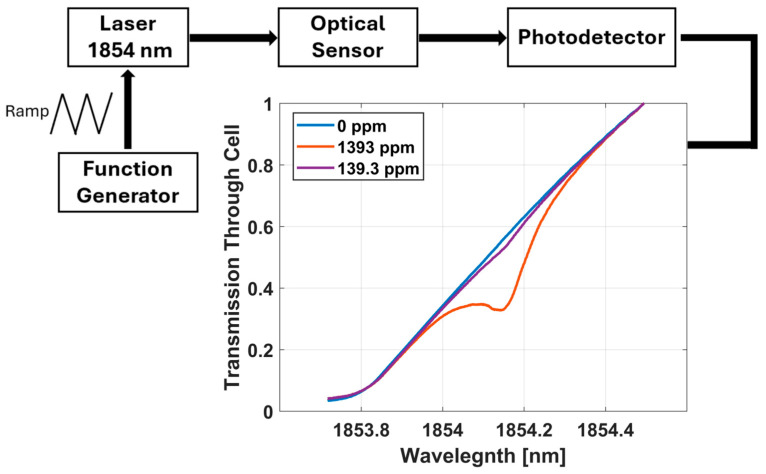
Direct absorption curves as monitored with TDLAS. The technique is limited at low concentrations as seen by the small transmission dip 139.3 ppm.

The input of the optical sensor uses a low power, narrowband laser (Eblana Photonics, Dun Laoghaire Co., Dublin, Ireland, modelEP1843-0-DM-DX1-FA) centered at 1854 nm to match the selected NIR absorption band of water vapor. A 50 µm core multimode fiber with a GRIN lens collimator is used as the input to the sensor (Thorlabs Incorporated, Newton, NJ, USA, model 50-1550M-APC) and a 400 µm core multimode fiber is used as the output fiber (Thorlabs Incorporated, Newton, NJ, USA, model FT400EMT-CUSTOM). The transmission of light through the optical sensor is monitored with an InGaAs-amplified photodetector (Thorlabs Incorporated, Newton, NJ, USA, model PDA10DT). The measurement methods used to monitor water vapor are tunable diode laser absorption spectroscopy (TDLAS) and wavelength modulation spectroscopy (WMS). Shown in Figure 2, we accomplish TDLAS measurements by using a function generator to bias a laser over the water vapor absorption line at 1854nm. The light passes through our sensor, interacts with water vapor in the headspace, and is collected by a photodetector. The spectrum produced shows a dip in transmission. When the transmission spectra are translated into absorbance, the resulting peak is proportional to the concentration of water vapor present in the system.

**Figure 3:**
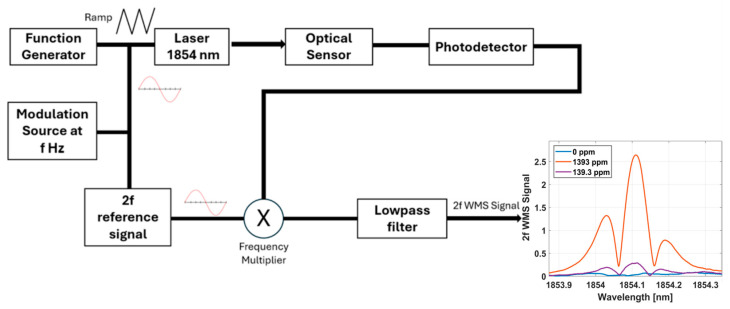
Implementation of WMS increases the limit of detection and expands the dynamic range of the sensor.

The strength of water vapor absorption lines in the NIR are relatively weak compared to the mid-infrared (MIR). This causes NIR sensors that use TDLAS to be limited at low concentrations (see plot, Figure 2). To enhance the limit of detection, WMS is applied in tandem by superimposing a high frequency sine wave onto the slower TDLAS ramp function. The modulated signal passes through the sensor, interacts with the analyte species, and is monitored with a photodetector. The signal is demodulated at twice the modulation frequency to produce a 2f WMS curve (shown in Figure 3). The same 139.3 ppm curve which was near the limit of detection with TDLAS reveals a measurable amplitude change with WMS. TDLAS and WMS are implemented with a custom LabVIEW program that drives and modulates signals from a data acquisition unit (National Instruments, Austin, TX, USA, model NI USB-6363, X Series DAQ (32 AI, 48 DIO, 4 AO). These two techniques are complementary to one another and can be executed simultaneously to increase the overall dynamic range of the sensor [15].

Once the optical alignment is established, the sensor is calibrated to properly translate units of absorbance into water vapor concentration. Bronkhorst (Sunnyvale, CA, USA) mass flow controllers are used to manipulate the flow of liquid water (model number L01-RAD-11-0) and dry nitrogen gas (model number F-201CV-5K0-RAD-11-V) into a controlled evaporator mixer (model number W-102A-111-K). The humidified nitrogen from the output of the mixer is infused into a sealed vessel where optical measurements take place. Software developed by Bronkhorst, Incorporated (Flowsuite 9.17.183: FlowDDE, FlowPlot, FlotView), records the output flow rate from each controller in real-time, which designates an upper and lower bound of the anticipated water concentration at a given setpoint during calibration. Each setpoint is typically maintained for at least an hour to allow the humidity within the headspace to equilibrate. An example of two calibration curves using TDLAS and WMS are shown in Figure S2, where the limit of detection of 7.5 ppm is experimentally demonstrated [15].

**Figure 4:**
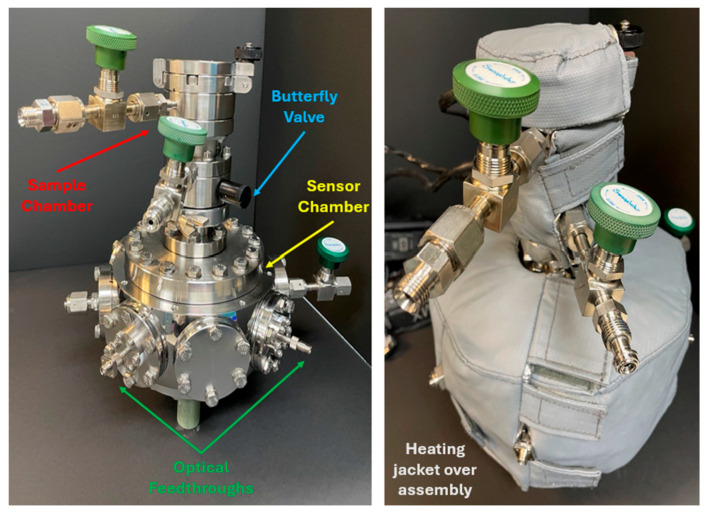
Images of the mechanical assembly with (**right**) and without (**left**) the heating jacket. More details are provided in Figure S3.

Our group produced a custom mechanical assembly that embeds the optical sensor during material outgassing trials. The assembly, shown in Figure 4, has two main chambers. The bottom chamber houses the optical sensor (termed “sensor chamber”) and the top chamber houses material coupons to be studied (termed “sample chamber”). The sensor chamber features an octagonal design with ConFlat flanges on each facet to promote versatility and meet the user’s needs (model number MCF600-SphOct-F2C8, Kimball Physics, Wilton, NH, USA). Our configuration (shown in Figure S3a) has flanges with two optical feedthroughs located at 45° from one another, a gas inlet and outlet for purging located at 180° from each other, two transparent windows to view inside during experimentation, and two blank flanges. The sample chamber (shown in Figure S3b) connects to the top of the sensor chamber. It contains a gold wire basket (shown in Figure S3c) to suspend samples in the headspace, a gas inlet and outlet for purging, and a butterfly valve that acts as a temporary spatial barrier between the sample and sensor chamber. The gold wire basket was made of 304 stainless-steel with a 300 mesh across the surface to promote gas exchange between the sample and sensor. The basket was electroplated with gold to passivate the surface and baked out before use. The butterfly valve is a crucial component to the system to ensure the sensor chamber maintains a constant baseline while samples are added into the system. The fully assembled vessel has a custom heating jacket wrapped around the outside during experimentation to control the temperature (supplied by TGM, Incorporated, Richardson, TX, USA). The total internal volume of the full assembly was determined to be 1218.5 cm^3^ by conducting a negative space infill in SolidWorks version 2024sp0.2.

Sylgard-184, a common silicone elastomer, was used as a case study to test the viability of the sensor and experimental system. The material’s biocompatibility (chemically inert and nontoxic), hydrophobicity, and simple fabrication procedure have led to adoption in many biomedical devices [16,17]. Two Sylgard-184 coupons with largely different volumes, were prepared from an off-the-shelf fabrication kit from Dow Corning (Midland, MI, USA). Briefly, the resin/curing agents in the kit consist of vinyl end-capped oligomeric dimethyl siloxane chains cross-linked with methyl hydrosiloxane reinforced with trimethylated silica, and a platinum catalyst. A 10:1 volume ratio of resin to curing agent was mixed and poured into cylindrical molds. After removing trapped air bubbles, the molds are cured at room temperature for 12 h followed by an additional 24 h vacuum treatment to eliminate residual volatile components. The cured samples are cut to specific geometries using a razor blade. More details on the fabrication procedure can be found in previously published work [18].

Prior to material testing, the assembly is heated to 56.75 °C and purged with dry nitrogen at 200 sccm for at least 15 h to ensure dry starting conditions. Sylgard-184 samples are preconditioned in a separate environmental chamber (Espec SH-242) at 70 °C and 70% relative humidity (RH). Once the system is fully prepared with dry nitrogen and the sample is fully preconditioned, the butterfly valve, the gas inlet, and gas outlet are closed on the sensor chamber. The lid for the sample chamber is opened and the Sylgard-184 is quickly dropped into the gold basket. An additional ten second purge of the sample chamber is conducted at 4000 sccm to drive out any excess water vapor that may have entered the system from the outside environment during the sample transfer. At this point the gas inlet and outlet on the sample chamber are closed and the butterfly valve is opened to allow outgassed water vapor from the sample to diffuse into the sensor chamber. Three separate trials were conducted on both Sylgard-184 samples to monitor the water vapor outgassing profile. The optical sensor recorded absorbance data every minute for 1200 total minutes.

Our experimental data were compared to the reaction, sorption, and transport model (ReSorT) [19,20,21]. In ReSorT, a sorption–diffusion model is used to describe water vapor transport and sorption dynamics based on three modes: a Henry’s mode corresponding to mobile water molecules diffusing through the material; a Langmuir mode, representing water molecules stuck at the surface or in a secondary phase in the material; and a pooling mode representing water molecules clustering in small regions in the material’s microstructure such as pores. The Langmuir and pooling modes correspond to immobile water vapor molecules as there is no mobile diffusion of these modes through the material. However, both Langmuir and pooling modes have kinetic sorption coefficients which dictate the rate of Langmuir adsorption and clustering components, respectively. The differential equations for Henry’s diffusion, Langmuir adsorption, and pooling due to capillary condensation are shown in Figure S4. Comparing our experimental data to ReSorT modeling predictions helps verify and validate new outgassing protocols that use our in-situ optical sensor. The ReSorT model also provides predictions which inform our experimental execution, including predictions of the minimum sample preconditioning time and of the optimal chamber purging time. The experimental data on the other hand offers the opportunity of further model validation and optimization.

## 3. Results

**Figure 5:**
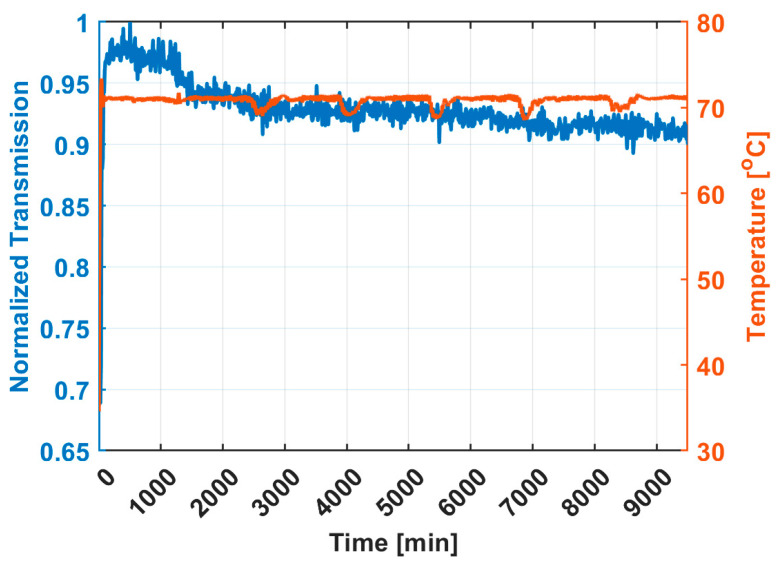
The transmission through the optical sensor normalized to the max transmission value (left axis) and temperature (right axis) as a function of time.

The material outgassing experiments were performed at 56.75 °C. Two temperature studies were conducted to ensure the optical sensor can operate at thermal elevation. The first study placed the optical sensor in a sealed stainless-steel container while heated to 71 °C for 9500 min. Throughput measurements were taken by monitoring the photodetector signal from the sensor with an input wavelength away from the water vapor absorption line. The results of the isothermal hold test are shown in Figure 5. In the first 500 min of heating, the cell transmission quickly spikes to its maximum value (normalized transmission of 1). From t = 1500 min to t = 9500 min, the transmission maintained an average normalized transmission value between 90% and 95% of the maximum transmission recorded during the experiment. The small deviations suggest that the alignment of the optical sensor is not impacted by heated conditions for long periods of time. A notable observation is that the sensor throughput increases in the initial stage from 0.68 to 1 during the transition from ambient temperature to the 71 °C setpoint. It is expected that heating and cooling will cause the optical fibers to expand and contract and thus alter the throughput of the sensor. However, a more rigorous thermal test would include a time-dependent temperature profile to ensure the sensor can remain stable after numerous heating and cooling cycles. A second thermal experiment introduced a time-dependent thermal ramp profile that ranged from −54 °C to 71 °C. The cell transmission versus temperature data can be found in Figure S5. The temperature range introduced in this experiment is much more extreme than what is anticipated in a typical material outgassing trial. However, a key finding from this test is that the cell signal does not ever drop to 0 V, suggesting that the alignment of the optical path is stable over a large operating temperature range. During a material outgassing trial, the signal variability seen in Figure S5 can be taken care of through normalization of either the direct absorption or WMS response. By accounting for proper temperature calibration, these results indicate that the sensor can operate under a large thermal range without high variability and is suitable as a real-time, in situ sensor.

**Figure 6:**
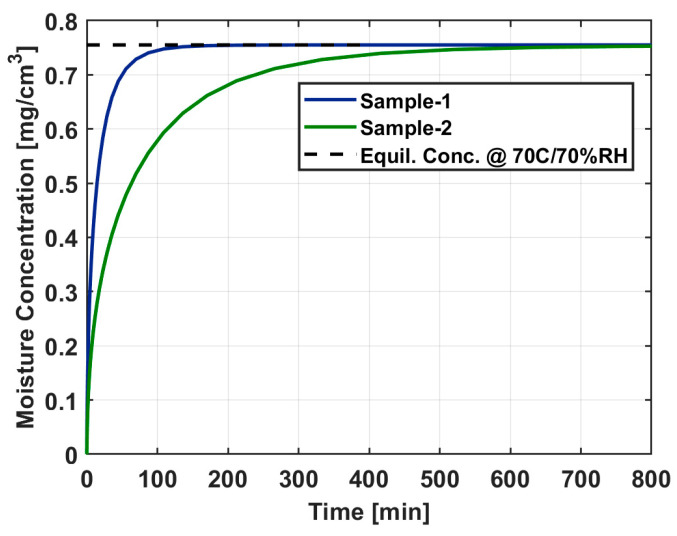
Total uptake time needed to reach an equilibrium concentration at 70 °C/70% RH for the two Sylgard-184 samples used in this study, using the ReSorT model.

Two geometries of Sylgard-184 samples were studied in this study. Sample-1 resembled a near-perfect cylinder and had a diameter of 21.21 mm and height of 6.09 mm for a total volume of 2.15 cm^3^ and a mass of 2.30 g. Sample-2 was not precisely machined and was included in this study to feature a more complex cylindrical geometry. The diameter remained consistent with Sample-1; however, the nonuniform height) led to larger uncertainty in the model. Based on caliper measurements along the perimeter of the sample, the average height of Sample-2 was calculated to be approximately 27.43 mm and had a mass of 9.86 g. The sample dimensions were imported and digitally meshed into the ReSorT model which was used to determine the total time needed for Sample-1 and Sample-2 to fully uptake water vapor at 70 °C and 70% relative humidity and the results are displayed in Figure 6. Sample-1 requires at least 150 min to reach the equilibrium concentration and Sample-2 requires at least 650 min to reach the equilibrium (>99%) concentration, due to an increase in volume. Both samples were preconditioned overnight for at least 900 min before an outgassing measurement to ensure full moisture sorption took place.

**Figure 7:**
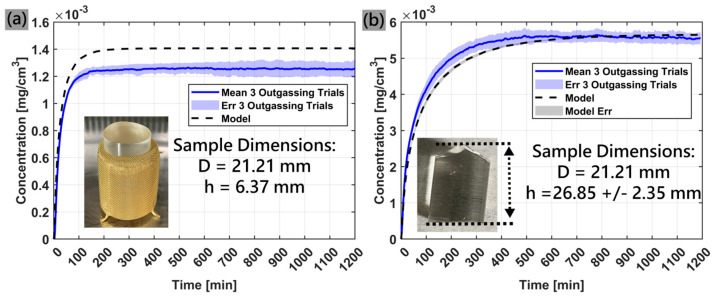
Outgassing profile of (**a**) Sample-1 and (**b**) Sample-2. Both plots include a comparison to the expected outgassing profile as determined by the ReSorT model.

Figure 7a shows the measured and simulated outgassing profile of Sample-1. The average equilibrium value of the experiment data was 1.26 × 10^−3^ ± 0.03 × 10^−3^ mg/cm^3^ while the model predicted a final steady state value of 1.41 × 10^−3^ mg/cm^3^. The measured and simulated outgassing profile of Sample-2 is shown in Figure 7b (showing the non-uniformity aforementioned). In this study, the model variance corresponds to the uncertainty in the volume of the sample which primarily affects the outgassing rate. The average equilibrium value of the experiment data was 5.55 × 10^−3^ ± 0.14 × 10^−3^ mg/cm^3^ while the model predicted a final steady state value of 5.65 × 10^−3^.

**Figure 8:**
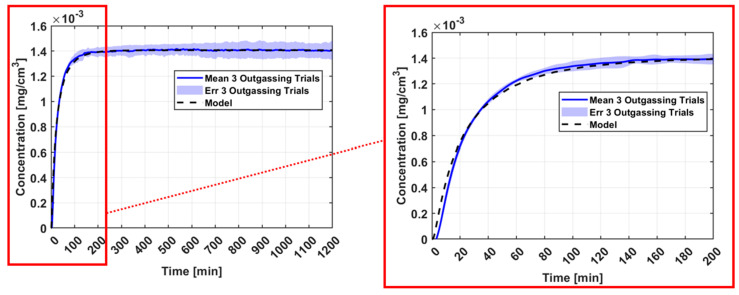
Comparison of the transient response between the model and experimental data after scaling the experimental data.

Another metric that is important to compare the experimental data to simulation is the transient response of the material which correlates to the overall shape of the curve. The transient time frame references the beginning of the experiment where the sorbed sample is placed in a dry environment and begins to outgas. A Pearson’s correlation coefficient between the measured and model data in Figure 7 was calculated to be 0.997 and 0.989 for Sample-1 and Sample-2, respectively, suggesting a near-perfect positive correlation between experiment and simulation [22]. However, to visualize the transient comparison of the simulated prediction of the material outgassing profile to the measured response, the experimental data curve from the Sample-1 experiment were scaled to match the final steady state concentration predicted by the model. This scaling will modify the experimental data such that the equilibrium value is equivalent to the simulated data (assumes no water vapor loss), thus allowing a proper comparison of the material’s initial reaction to the system. The scaling factor was determined by taking the ratio of the equilibrium model value to the equilibrium experimental data value. Scaling the experimental data by a factor of 1.12 produced the profile shown in Figure 8. Here, the transient profile in the first 200 min of measurement is emphasized which shows an overlapping match between model and data, suggesting the experiment properly measured the diffusivity of the material.

## 4. Discussion

The experimental data measured, on average, approximately 10.6% and 1.77% less water vapor than the model’s predictions for Sample-1 and Sample-2, respectively. To quantify the discrepancy between model and experiment, we used the outgassing measurements to compare the total water measured in the chamber at T_final_ to the anticipated water in the sample at T_0_. If we assume ideal conditions (no water loss), then the total amount of water vapor in the system should be equivalent to the amount of water vapor sorbed by the sample. Figure 5 suggests that a properly preconditioned sample of Sylgard-184 will saturate at 0.755 mg of water per unit volume of Sylgard in cm^3^. For our samples, this equates to 1.70 mg and 7.16 mg of water for Sample-1 and Sample-2, respectively. To make these values comparable, we scaled the total amount of water by the respective mass of each sample to yield 0.738 and 0.726 mg of water per g of Sylgard-184.

We compared these values with the amount of water measured in the system (from the equilibrium values in Figure 7) scaled by the effective chamber volume which accounts for the volume in the chamber occupied by the sample. We also considered that the Sylgard-184 sample will have residual water vapor inside the sample at T_final_ since the humidity inside the chamber increases over time. Adding these two components together yields an experimental calculation of 0.679 and 0.742 mg of water per g of Sylgard-184 for Sample-1 and Sample-2, respectively. Table S1 shows the calculation of each variable considered in this analysis

Based on these estimations, there is a reasonable amount of water loss in the experimental data that uses a real-world, nonideal system. For example, there is anticipated water vapor loss from the sample during the transfer window and during the initial ten second purge. The purging is a necessary step to ensure any ambient humidity that entered the system during sample transfer is removed before the measurement begins. Our results suggest that a small fraction of the samples’ overall preconditioned water content is desorbed to the atmosphere during sample transfer and chamber purging. Secondly, the complex volume of the outgassing chamber is likely a slight overestimation. The volume of the chamber was determined using a negative space infill in SolidWorks, which does not account for volume occupied by the sensor or optical fibers. This is likely to explain why the experiment for Sample-2 yields a slightly higher concentration of water on a per mass basis of Sylgard-184 compared to the model.

**Figure 9:**
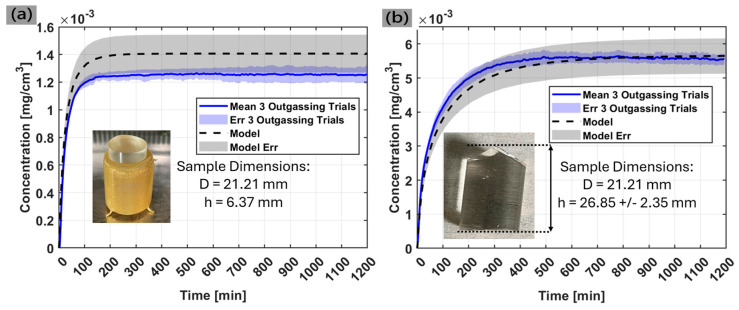
Outgassing profile of (**a**) Sample-1 and (**b**) Sample-2. Both plots include the model comparison with 10% error in the calibration parameters due to lot-to-lot variation.

Apart from the water loss during sample transfer and chamber purging, there is also an inherent uncertainty in the model’s prediction that is not factored into the data in Figure 7. The model uses parameters from Sylgard-184 that were determined from in situ gravimetric dynamic vapor sorption experiments in an IGAsorp developed by Hiden Isochema, Warrington, UK. In these trials, we observed approximately 10% variance in the mass uptake and outgassing of Sylgard-184 simply due to lot-to-lot variations across samples [8,23]. Figure 9 shows the model and experiment outgassing profiles with the error of the model included. Furthermore, the model parameters have been optimized using the total moisture content of the sample, rather than a distribution of moisture concentration as a function of space over the sample domain. So, multiple combinations of Henry’s, Langmuir, and pooling modes can produce the same total moisture content. Although we try to minimize this common issue by making sure that each of these modes are physically consistent, there can still be small variations in the model parameters.

A major advantage of in situ analysis is being able to monitor the initial outgassing profile during material desorption studies. Sample-1 features well-machined dimensions which give confidence in the model results that predict outgassing in ideal conditions. Due to the well-defined volume, we demonstrated the transient response of the experimental measurement matches the model’s prediction extremely well. This gives confidence that if a sample has well known dimensions, the experiment setup can successfully measure a material’s diffusivity and capacity of the material to sorb and desorb water vapor. Our measurement of Sample-2 showed the sensor measured a slightly faster uptake in moisture concentration than the model prediction. This indicates that the uncertainty in the exact sample dimensions leads to greater error in the transient prediction. For example, the simulation for Sample-2 imports the material into the ReSorT model as two different perfect cylinders, one with a minimum expected height and one with a maximum expected height based on caliper measurements of the real sample. While this should yield an upper and lower bound for the real sample volume, it may not be the only contributing factor in predicting vapor desorption from the sample. The nonuniform cut on one side of the sample (shown in the Figure 7b inset) shows an irregular spike pattern on one end of the cylinder. These spikes are expected to increase the sample surface area compared to a perfect cylinder which is not accounted for in the model. Since water vapor can diffuse out from any open interface along the sample’s surface, it is reasonable to measure a faster vapor desorption from the sample than what the model predicts. The results from this study indicate future work where the model can be improved, such as digitally reconstructing irregular sample geometry to precisely match real sample volume and surface area.

Finally, we put our sensor performance in the context of current state of the art multipass sensors focusing on two key metrics used for industrialized optical sensors designed for in situ monitoring: sensor size and sensitivity. Wang et al. recently demonstrated a novel prism array multipass NIR sensor targeting water vapor [24]. The authors specified an optical path length of 1.74 m and a theoretical limit of detection of 7.09 ppm based on the noise of the system. This aligns well with our optical path length of 1.83 m and our measured detection limit of 7.5 ppm; however, the total footprint of our sensor is 3.93 times smaller. Others have sought sensitivity enhancement by targeting stronger absorption bands in the mid-infrared, leading to research for multipass absorption cells using quantum cascade lasers [25,26]. While the sensitivity enhancement has shown to be an attractive research thrust, the high cost, large power consumption, and bulky instruments necessary to operate the systems limit their ability to act as an embedded diagnostic tool. Additionally, due to the rising interest in optical sensor technology, multipass cells have also started to gain a commercial presence. Perhaps the most advanced are the compact multipass gas cells designed for absorption spectroscopy recently offered by Thorlabs, Incorporated. Their version of the White cell design (model number MGC3C-P01) specifies an optical path length of 3.2 m with a total volume of 370.35 cm^3^. The volume, which is more than a three-fold increase compared to our sensor, is elevated further when one considers the input beam is external to the system and requires additional cage mounts to be inserted. To date, the performance of these commercial sensors remains unclear.

## 5. Conclusions

In this study, we have demonstrated several novel findings. First, we have designed and constructed a low-profile White cell sensor that can measure water vapor over a large dynamic range. An all-mechanical layout was implemented to ensure the sensor is inert and has robust thermal stability. Second, the optical sensor was embedded into a homemade assembly using off-the-shelf vacuum chamber components. We then devised a general experimental methodology that can be used for desorbed gas constituents from material coupons. Using these protocols, we demonstrated real time, in situ water vapor outgassing measurements from two Sylgard-184 samples that were shown to have excellent repeatability between trials. Finally, we corroborated the functionality of the sensor and the experimental methodology with a comparison to simulations from the ReSorT model. This dataset along with the comparison with simulations builds confidence in our experimental procedure and methodology for measuring the outgassing profile of sorbed samples. Comparing the real-world measurements with an ideal simulation provides a feedback loop where both systems inform one another and promote iterative optimization. In the future, this study can be expanded upon by beginning trials with more complex stacks of materials. Furthermore, the concept of the multipass design also has the flexibility to be integrated with broadband instruments monitor multiple species simultaneously.

## Data Availability

The data supporting the findings of this study are available from the corresponding author upon reasonable request, subject to privacy restrictions.

## Supplementary Materials

The following supporting information can be downloaded at: https://www.mdpi.com/article/10.3390/s25123824/s1.
