# Supplementary material for: Fiber-Coupled Multipass NIR Sensor for In Situ, Real-Time Water Vapor Outgassing Monitoring
Source: Sensors (Basel). 2025 Jun 19;25(12):3824. doi: 10.3390/s25123824 (PMC12197183; doi:10.3390/s25123824)
Supplement: Supplementary file 1 [file sensors-25-03824-s001.zip › sensors-3676451-supplementary.pdf]

Supplementary Images

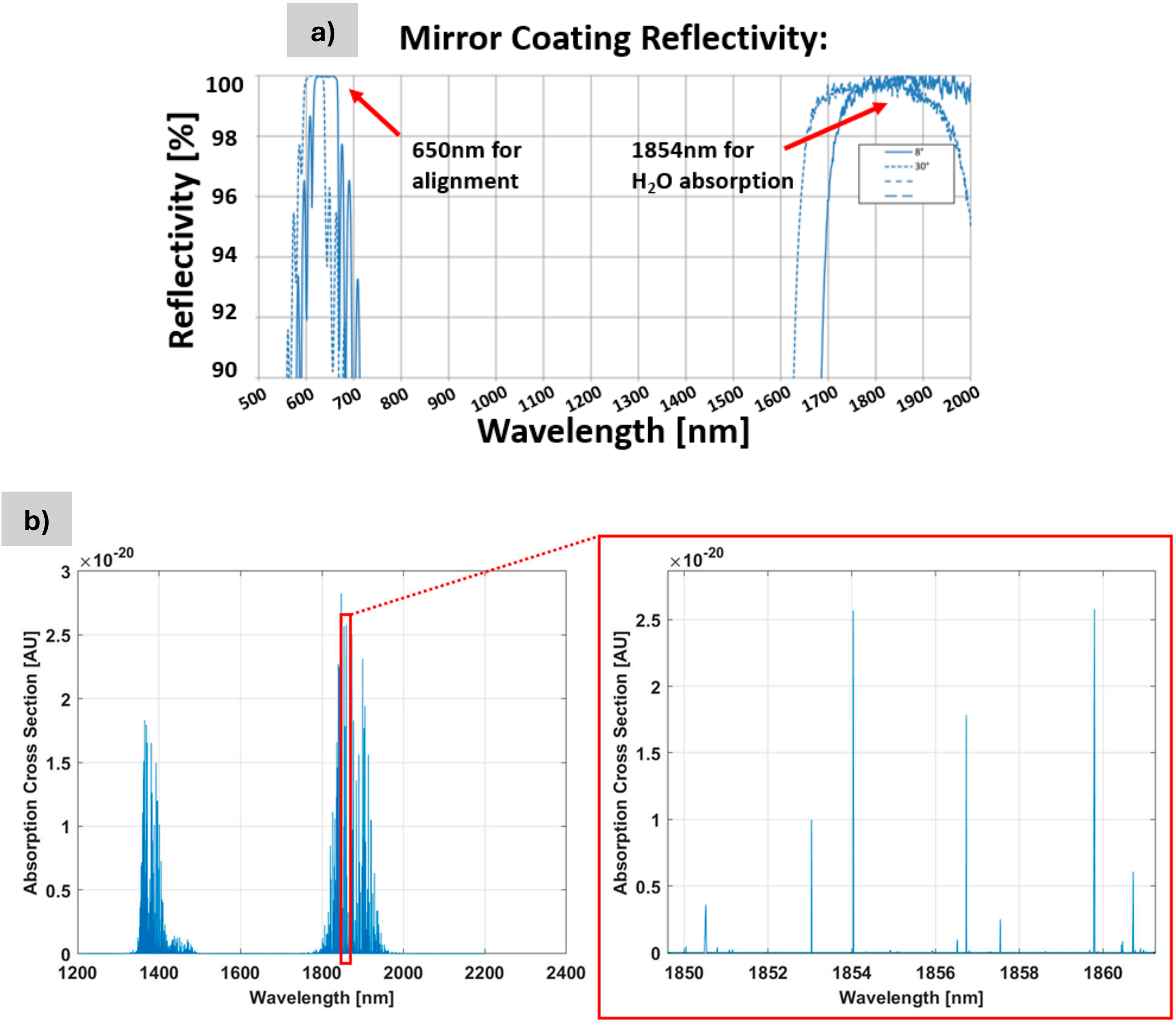

Figure S1. a) The mirror reflectivity after dielectric coating is optimized for 650 nm for alignment with a visible laser and 1854 nm for water vapor absorption. b) NIR water vapor absorption spectra from HITRAN.

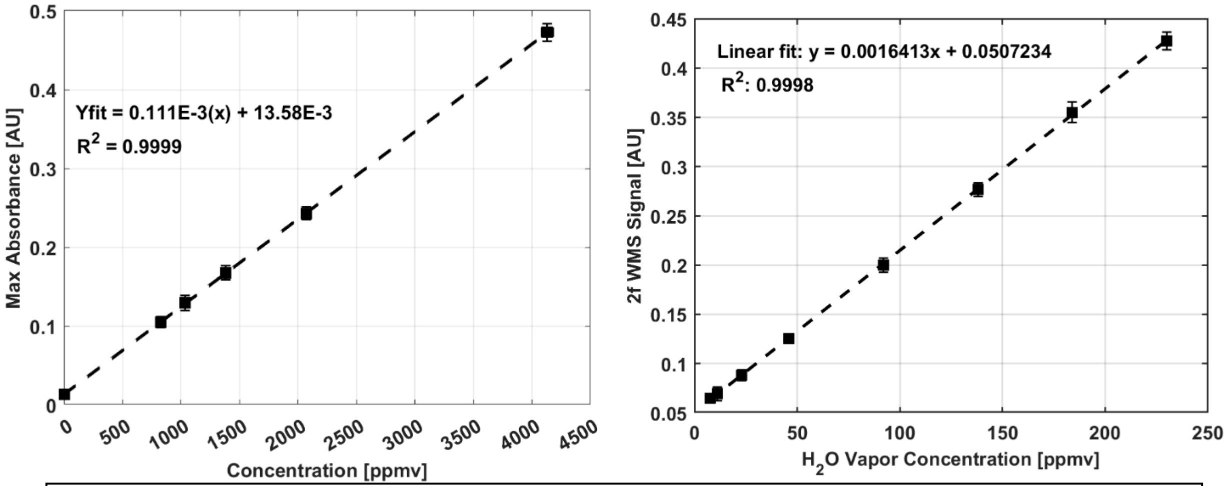

Figure S2. Calibration curves using TDLAS (left) over a high concentration range (up to 4200 ppmv) and WMS (right) over a low concentration range (down to 7.5 ppmv)

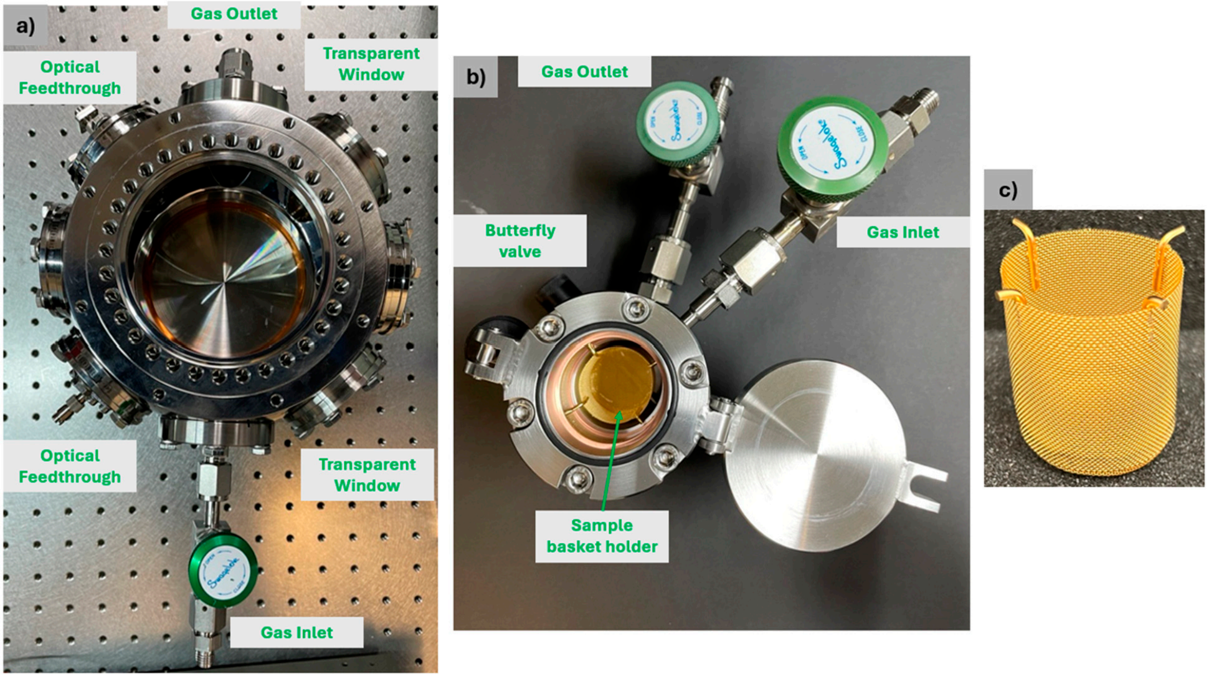

Figure S3. The sensor chamber (a) sample chamber (b) and sample basket holder (c) with relevant parts labelled.

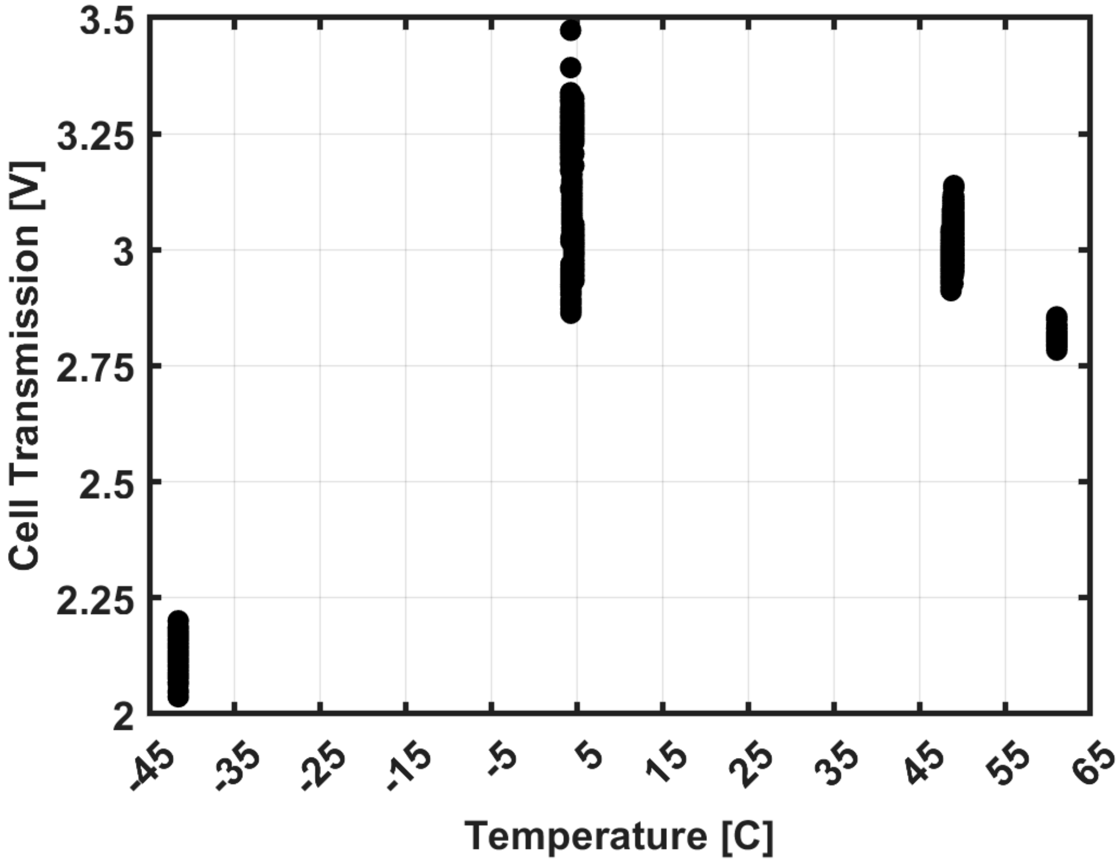

Figure S4. The cell transmission over a large temperature range (-43°C to 63°C). The temperature was cycled between 5°C and 47°C many times leading to a larger standard deviation.

	Sample Volume	Sample Mass	Water in Sample after Precondition	Measured Outgassing Equilibrium Value	Effective Chamber Volume	Residual Water in Sample at Equilibrium*	Total Water in the System	Total Water in the System on a Per Mass Basis
**Sample-1**	2.25 cm^3	2.30 g	0.738 mg (water) per g (Sylgard-184)	1.26 x 10^{-3} mg/cm^3	1216.29 cm^3	0.029 mg	1.56 mg	0.679 mg (water) per g (Sylgard-184)
**Sample-2**	9.49 cm^3	9.86 g	0.726 mg (water) per g (Sylgard-184)	5.55 x 10^{-3} mg/cm^3	1209.05 cm^3	0.608 mg	7.32 mg	0.741 mg (water) per g (Sylgard-184)

Table S1. Relevant metrics used in the analysis that compares water measured in the system at T_{final} to the total amount of water anticipated to be in the sample after preconditioning. The residual water in the sample is determined by the ReSorT model which calculates the sorption of water at the humidity measured by the sensor at T_{final}^{*}. Multiplying the measured outgassing equilibrium value with the effective chamber volumes and adding with the residual water in the sample at equilibrium yields the total amount of water in the system.
